# Efficacy and hepatic complications of three endovascular treatment approaches for delayed postpancreatectomy hemorrhage: evolution over 15 years

**DOI:** 10.1186/s42155-019-0077-x

**Published:** 2019-10-22

**Authors:** Yu-Chien Chang, Kao-Lang Liu, Yu-Cheng Huang, Po-Ting Chen, Yu-Wen Tien, Yen-Heng Lin, Yeun-Chung Chang

**Affiliations:** 1 Department of Medical Imaging, National Taiwan University Hospital and National Taiwan University College of Medicine, No. 7, Chung-Shan South Rd, Taipei, 100 Taiwan, Republic of China; 2 Department of Medical Imaging, National Taiwan University Cancer Center, National Taiwan University College of Medicine, Taipei, Taiwan, Republic of China; 3 Department of Surgery, National Taiwan University Hospital and National Taiwan University College of Medicine, Taipei, Taiwan, Republic of China

**Keywords:** Pancreaticoduodenectomy, Delayed postpancreatectomy hemorrhage, Transarterial embolization, Covered stent

## Abstract

**Background:**

Delayed postpancreatectomy hemorrhage (PPH) is a fatal complication caused by arterial erosion. This study reports a single-center experience of managing delayed PPH with different endovascular treatment approaches.

**Methods:**

We reviewed the data of patients who had delayed PPH due to hepatic artery or gastroduodenal artery stump perforation and underwent endovascular treatment between 2003 and 2018. We categorized endovascular treatment approaches involving hepatic artery sacrifice, superselective pseudoaneurysm embolization with hepatic artery preservation, and covered stent placement. Technical success rates, hemorrhage recurrence rates, major and minor hepatic complication rates, and 30-day and 1-year mortality rates were assessed.

**Results:**

A total of 18 patients were reviewed; 11 (61%), 4 (22%), and 3 (17%) delayed PPH cases were managed through hepatic artery sacrifice, superselective pseudoaneurysm embolization, and hepatic artery stenting, respectively. Multidetector computed tomography was performed in 14 (78%) patients. The technical success rate was 100%. The overall hemorrhage recurrence rate was 39%, with superselective pseudoaneurysm embolization having a 100% hemorrhage recurrence rate—much higher than that of hepatic artery sacrifice or stent graft placement. The overall major and minor hepatic complication rates were 56% and 83%, respectively. The overall 30-day and 1-year mortality rates were 11% and 25%, respectively. The 30-day and 1-year mortality rates and minor and major hepatic complication rates were similar in each group.

**Conclusion:**

Hepatic artery sacrifice is more effective than superselective pseudoaneurysm embolization in the management of delayed PPH. Covered stent placement may be a reasonable alternative treatment to hepatic artery sacrifice.

## Background

Pancreaticoduodenectomy, a complex surgical procedure for resecting tumors or ameliorating inflammation in the periampullary region, is performed as either a classic pancreaticoduodenectomy (Whipple procedure) or a pylorus-preserving pancreaticoduodenectomy (PPPD). Common complications associated with this procedure include anastomotic leakage, infection, and hemorrhage (Bhosale et al. 2013; Raman et al. 2013; Malleo and Vollmer Jr. 2016). Postpancreatectomy hemorrhage (PPH) is observed in less than 10% of patients but is responsible 11%–38% of the associated deaths (Puppala et al. 2011). PPH can be classified as early or delayed PPH according to the definition of the International Study Group of Pancreatic Surgery (Wente et al. 2007). Delayed PPH is defined as hemorrhage occurring more than 24 h postoperatively, and its etiology is related to postoperative leakage due to anastomotic failure and localized inflammation. Continued inflammation can lead to splanchnic vessel wall erosion, thus explaining delayed PPH (Hasegawa et al. 2017).

Relaparotomy, endoscopy, and endovascular treatment (EVT) have been described as treatments used for managing delayed PPH. Endoscopy plays a limited role, and relaparotomy is indicated for conditions of insufficient hemostasis despite endoscopy or EVT (Khalsa et al. 2015). Strategies for managing delayed PPH have shifted from surgery toward EVT over the past decade (Zhang et al. 2011; Adam et al. 2014; Asai et al. 2015; Khalsa et al. 2015; Zhou et al. 2017; Biondetti et al. 2019). The common hepatic artery and gastroduodenal artery (GDA) stump are the most common culprit vessels (Hasegawa et al. 2017). Different EVT approaches, including hepatic artery sacrifice and superselective pseudoaneurysm embolization, have been described (Hur et al. 2011; Stampfl et al. 2012). Recent studies have reported that covered stent grafts are effective for both managing delayed PPH and preserving hepatic artery flow (Hankins et al. 2009; Ching et al. 2016). Despite the various alternatives, the hepatic complication rates of these treatment approaches have been variable in thus far. The mid-to-long-term clinical outcome data of these strategies remain scarce.

In the present study, the short- and mid-term clinical outcomes of three EVT approaches, namely hepatic artery sacrifice, superselective pseudoaneurysm embolization with hepatic artery preservation, and covered stent placement, for managing delayed PPH were explored.

## Materials and methods

### Patients

We retrospectively reviewed the databank at National Taiwan University Hospital for 2003–2018. This study was approved by the institutional review board of the hospital. We searched for electronic medical records of patients who received the Whipple procedure or PPPD. Patients who had delayed PPH and underwent EVT were included. Patients were excluded if (1) the culprit vessel was not branched from the common hepatic artery or (2) the clinical or image data were missing or were insufficient for analysis. In total, 19 patients had delayed PPH and underwent EVT. Because one patient with a splenic artery pseudoaneurysm after the Whipple procedure was excluded, 18 patients were included in the final analysis.

### Clinical management and data assessment

Hemorrhage was detected by the presence of either sentinel bleeding, defined as blood in the abdominal drain, or hematemesis and melena. All patients presented with delayed PPH, defined as hemorrhage occurring more than 24 h postoperatively. Most patients underwent multidetector computed tomography (MDCT) angiography for culprit lesion detection.

Clinical data, including age, sex, pathologic diagnosis, coagulation profile, clinical presentation, and onset time of bleeding after surgery, were obtained from the available medical records. On the basis of the International Study Group for Pancreatic Fistula (Bassi et al. 2017), we defined pancreatic leakage as an amylase concentration greater than three times the upper limit of normal serum amylase concentration in the drain tube after postoperative day 3. Moreover, we defined coagulopathy as a serum platelet count of less than 50,000 × 10^6^/L or an international normalized ratio of > 1.5 (Hasegawa et al. 2017).

### Endovascular procedures

After a vascular sheath was introduced through either the right or left common femoral artery, a 4- or 5-Fr angiographic catheter was navigated at the celiac artery for angiography, and angiographic findings regarding the culprit lesion were identified as either contrast spillage or a pseudoaneurysm. Three EVT strategies, namely destructive, superselective, and constructive approaches, were applied on the basis of the MDCT findings, available embolization materials, and duty doctors’ experience.

### Superselective approach

**Fig. 1:**
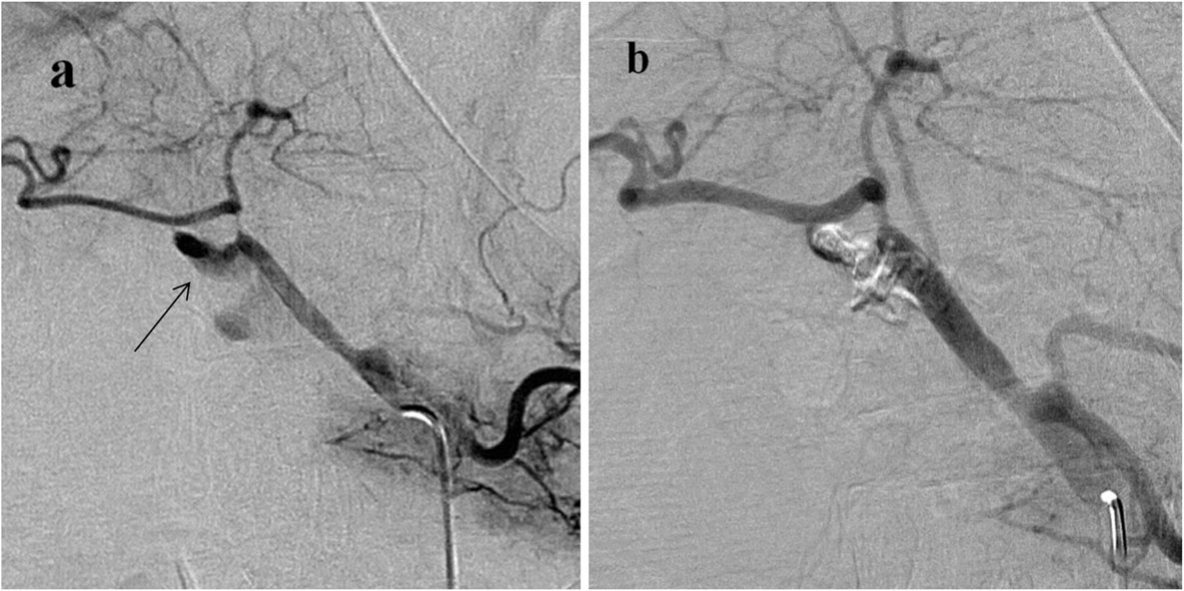
Example of superselective embolization conducted on a 69-year-old man with delayed PPH 17 days after the classic Whipple procedure for cancer of the ampulla of Vater. **a** Celiac angiogram showing active bleeding (black arrow) at the common hepatic artery. **b** Superselective embolization with 40% N-butyl cyanoacrylate (NBCA)–lipiodol mixture through a 1.7-Fr microcatheter (Excelsior SL-10; Boston Scientific, Fremont, CA, USA) was performed on the bleeding site. Postembolization angiogram showed preservation of the proper hepatic artery. Technical success was achieved initially

This approach, was frequently employed at the hospital between 2005 and 2008, involved the application of embolization to the culprit lesion (Fig. 1). Hepatic artery patency was preserved intentionally. A microcatheter was typically navigated into the lesion, which was either a pseudoaneurysm or active bleeding site, and pushable coils (Cook, Bloomington, IN, USA) or a 40%–50% N-butylcyanoacrylate (NBCA)–lipiodol mixture were used.

### Destructive approach

**Fig. 2:**
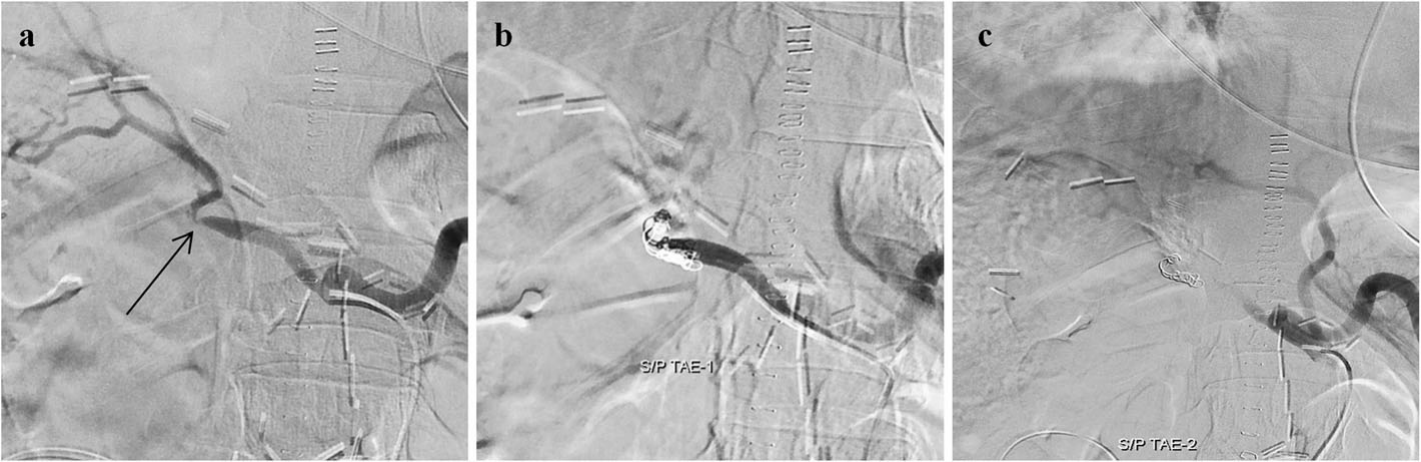
Example of destructive approach performed on a 65-year-old man with delayed PPH 17 days after the Whipple procedure for solid pseudopapillary neoplasm of the pancreas. **a** Celiac angiogram showed a segmental, irregular, and narrow proper hepatic artery at the GDA stump with associated beaded protrusions (black arrow). **b** Pushable coils (Cook, Bloomington, IN, USA) were deployed from the proper hepatic artery to the common hepatic artery by using the sandwich technique. **c** Postembolization angiography showed complete occlusion of the proper hepatic artery and collateral vessels to liver parenchyma from the left gastric artery

This approach has been employed at the hospital since 2003. The destructive approach involved hepatic artery sacrifice (Fig. 2). The catheter was navigated to the distal part of the culprit lesion, and pushable coils were deployed from the distal to the proximal part of the lesion (sandwich technique). When navigation to the distal portion was difficult, a 40%–50% NBCA–lipiodol mixture was used to occlude the proper hepatic artery or common hepatic artery from the proximal portion.

### Constructive approach

**Fig. 3:**
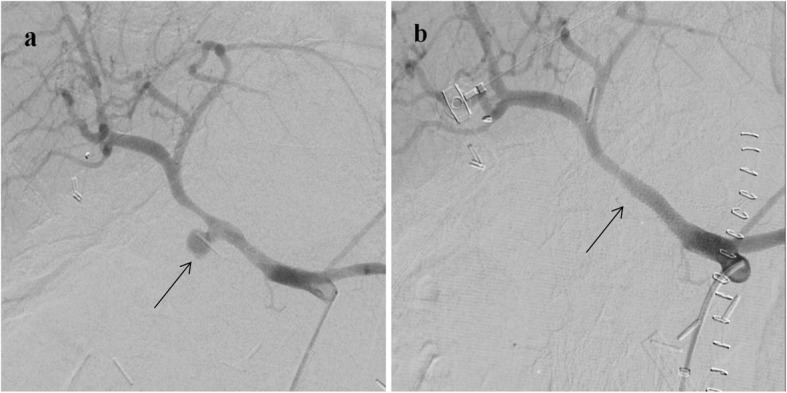
Example of constructive approach performed on a 65-year-old woman with delayed PPH 20 days after the Whipple procedure for ampulla of Vater tubulopapillary adenoma with focal high-grade dysplasia. **a** Celiac angiogram demonstrating a pseudoaneurysm GDA stump (black arrow). **b** After an 8-Fr-long sheath was placed at the celiac orifice, a 6 mm × 5 cm stent graft was deployed covering the pseudoaneurysm (black arrow). Postembolization angiogram showed complete exclusion of the pseudoaneurysm and patent hepatic artery

Covered stent placement has been performed at the hospital since 2016. A 45-cm, 6–8-Fr-long vascular sheath (*Cook Medical,* Bloomington, IN, USA) was placed at the celiac origin. After the distal intrahepatic artery was wired, a self-expandable polytetrafluoroethylene-covered stent (Viabahn; W. L. Gore and Associates, Flagstaff, AZ, USA) was navigated onto the lesion and deployed across the culprit lesion (Fig. 3). Poststenting angioplasty was not conducted routinely. Antiplatelet medications were not administered considering the current bleeding condition.

### Outcome assessment

Technical success was confirmed after a review of angiography after EVT. We defined technical success as the cessation of active contrast extravasation or nonopacification of a pseudoaneurysm. Hemorrhage recurrence rates, 30-day and 1-year mortality rates, and major and minor hepatic complication rates were recorded. Recurrent hemorrhage was defined as any evidence of bleeding, such as increased bloody drain or hematemesis, unstable vital signs necessitating the consultation of interventional radiologists, and evidence of bleeding in follow-up MDCT or angiography.

We defined major and minor hepatic complications according to the standards of the Society of Interventional Radiology (Sacks et al. 2003) and Hasegawa et al.’s (2017) study. Major hepatic complications included hepatic failure and hepatic abscess. Hepatic failure was defined as an increase in aspartate and/or alanine aminotransferase levels by 1000 U/L or an increase in the total bilirubin level by 10 mg/dL within 7 days after EVT. Hepatic abscess was defined as the combination of liver abscess observed in contrast-enhanced computed tomography (CT) and clinical signs and symptoms of infection. Minor hepatic complications involved abnormal hepatic function, defined as a temporary increase in the level of aspartate and/or alanine aminotransferase three times above the normal upper limit of the serum level within 14 days after EVT.

### Statistical analysis

Because of the small sample size, inferential statistical analysis could not be performed. Descriptive statistics related to the clinical data were obtained. Technical success, hemorrhage recurrence rates, 30-day and 1-year mortality rates, hepatic failure and abscess rates, and abnormal liver function rates of the three approaches are provided.

## Results

### Clinical characteristics and angiographic findings

Among the 18 selected patients, the mean age was 67 ± 11 years, and 10 (56%) patients were male. Malignancy was diagnosed in 10 (56%) patients. The average time of bleeding onset was 30.3 days (range: 7–170 days) postoperatively. The patient bleeding 170 days postoperatively had been discharged after the surgery, but he developed a recurrent intraabdominal infection and eventually experienced bloody vomiting. MDCT angiography was performed in 14 (78%) patients at the onset of bleeding. In 2003, two patients did not undergo CT angiography before EVT because we did not have MDCT then. The remaining two patients did not receive MDCT because their conditions were urgent or the duty radiologist directly decided to perform angiography. MDCT revealed active bleeding or pseudoaneurysm at the common hepatic artery or its branches in 11 patients. Pancreatic leakage and coagulopathy were observed in 14 (78%) and 4 (24%) patients, respectively. Detailed clinical data are presented in Table 1. The most frequent bleeding site was the GDA stump (67%). We observed three vascular anatomical variances. For EVT, 11 (61%), 4 (22%), and 3 (17%) delayed PPH cases were managed through hepatic artery sacrifice, superselective pseudoaneurysm embolization, and hepatic artery stenting, respectively. Angiographic and embolization details are presented in Table 2.

**Table 1:** Patient characteristics

Clinical variables	
Sex-number(%)	
Male	10 (56)
Female	8 (44)
Age - mean (SD)	67 (11)
Pathological diagnosis - number(%)	
Duodenal cancer	2 (11)
Bile duct cancer	2 (11)
Ampullar of Vatar cancer	3 (17)
Pancreatic cancer	2 (11)
Pancreatic head solidpseudopapillary neoplasm	1 (6)
^a^Other benign disease	8 (44)
Surgical method - number(%)	
Classic Whipple procedure	12 (75)
PPPD	6 (25)
Clinical findings of bleeding – number(%)	
Sentinel bleeding	8 (44)
Hematemesis or melena	9 (50)
No documented	1 (6)
Coagulopathy(*N* = 17) – number(%)	4 (24)
Postoperative day - mean (range)	30.3 (7–170)
CT performed before EVT – number(%)	14 (78)
Pancreatic leakage(*N* = 17) – number(%)	14 (82)

^a^Includes common bile duct (CBD) papillary hyperplasia, pancreatic head low-grade intraductal papillary mucinous neoplasm with low-grade dysplasia*2, CBD chronic inflammation, CBD tubulopapillary adenoma, chronic pancreatitis with pseudocyst formation, ectopic pancreas in the periampullary area and chronic inflammation, and ampulla of Vater tubulopapillary adenoma with focal high-grade dysplasia

**Table 2:** Angiography findings and other results

Variables	
Bleeding vessels – number (%)	
GDA Stump	12 (66)
Proper hepatic artery	1 (6)
Either proper hepatic artery or GDA stump	2 (11)
Common hepatic artery	2 (11)
Right hepatic artery	1 (6)
Angiographic findings – number (%)	
Pseudoaneurysm	10 (55)
Active bleeding	7 (39)
^a^No evidence of hemorrhage	1 (6)
Vascular anatomical variants – number (%)	3 (17)
Embolization material – number (%)	
Coil	6 (33)
Coil and NBCA	5 (28)
NBCA	3 (17)
^b^NBCA and intraarterial epinephrine	1 (6)
Covered stent	3 (17)
Embolization methods – number (%)	
Hepatic artery sacrifice	11 (61)
Superselective	4 (22)
Covered stent placement	3 (17)

^a^CT and angiography showed a small GDA stump and but we thought it was a normal postsurgical finding. 3 days later it ruptured and we embolized with pushable coils

^b^Epinephrine was diluated as 1: 1000 and slowly infused via microcatheter after NBCA injection

### Clinical outcomes of different EVT methods

Technical success was achieved in all patients. The overall hemorrhage recurrence rate was 39%. The major and minor hepatic complication rates were 56% and 83%, respectively. Major and minor hepatic complications occurred in all subgroups, with no specific EVT method being more effective than the other. The overall 30-day and 1-year mortality rates were 11% and 24%, respectively. Detailed clinical outcomes of each strategy are presented in Table 3.

**Table 3:** Efficacy and hepatic complications of three endovascular treatment approaches

	Total (*N* = 18)	Hepatic artery sacrifice group(*N* = 11)	Superselective embolization group (*N* = 4)	Covered stent group (*N* = 3)
Technical success - number(%)	18 (100)	11 (100)	4 (100)	3 (100)
Recurrent hemorrhage - number(%)	7 (39)	2 (18)	4 (100)	1 (33)
Hospital stay - mean (SD)	75 (53)	61 (49)	103 (66)	91 (53)
30-day mortality - number(%)	2 (11)	2 (18)	0 (0)	0 (0)
1-year mortality- number(%)	4 (24), *N* = 17	2 (20), *N* = 10	1 (25)	1 (33)
Major hepatic complications	10 (56)	6 (55)	2 (50)	2 (67)
Hepatic failure - number(%)	5 (28)	3 (27)	1 (25)	1 (33)
Hepaitc abscess - number(%)	6 (33)	4 (36)	1 (25)	1 (33)
Minor hepatic complication - number(%)	15 (83)	8 (73)	4 (100)	3 (100)

### Superselective approach

Four patients received the superselective approach. Three cases occurred before 2008 and one in 2016. The rebleeding rate was 100%, and in all cases, rebleeding occurred at the sites of first treatment. We managed the rebleeding by using either hepatic artery trapping (*n* = 3) or a covered stent (*n* = 1). Hepatic failure occurred in 1 (25%) patient. Liver abscess developed in 1 (25%) patient. All patients had minor hepatic complications. Moreover, 1 (25%) patient died 65 days after embolization due to recurrent bleeding and hepatic failure.

### Destructive approach

In total, 11 patients received hepatic artery trapping. The hemorrhage recurrence rate was 18% (*n* = 2), and both rebleeding sites were the same as the first treated locations. Two patients who had clinical symptoms of bleeding but showed negative angioplasty or CT angiography findings were considered to have no rebleeding. We used more coils at the rebleeding site for hemorrhage alleviation. Six (55%) patients had major hepatic complications, whereas eight (73%) had minor hepatic complications. Two (20%) patients died within 30 days after embolization. One patient died of a catheter-related blood stream infection and septic shock. Another patient had liver abscess before the EVT and died of multiple organ failures.

### Constructive approach

Three patients received covered stent placement to cover the bleeding GDA stump. The rebleeding rate was 33% (*n* = 1), and major and minor hepatic complication rates were 67% (*n* = 2) and 100% (*n* = 3), respectively. None of the patients died within 30 days after EVT, but one (33%) died within 1 year. Detailed analysis of the three cases revealed that one patient died of septic shock and liver abscess 131 days after stenting, despite short-term endovascular success. Another patient had liver failure, possibly due to a hematoma, which caused afferent loop syndrome, but this patient recovered subsequently and was lost to follow-up 273 days after embolization. The third patient had recurrent hemorrhage 151 days after stenting and survived longer than 1 year. This patient’s recurrent bleeding site was the proximal common hepatic artery, different from the initial treated site. This location necessitated combined destructive and constructive approaches for further management. We did not perform balloon angioplasty, and no endoleak was noted in follow-up CT.

## Discussion

In the present study, the superselective approach was associated with a much higher hemorrhage recurrence rate than was the destructive or constructive approach, suggesting its unreliability. Moreover, hepatic complication occurred in each treatment subgroup regardless of whether the patients received stent placement. Finally, no specific treatment approach was more efficient than the other in terms of 30-day and 1-year mortality rates. Multiple factors affected the prognosis, such as the surgery or the patients’ underlying conditions.

The superselective approach is intuitively attractive because it preserves hepatic arterial supply. Stampfl et al. (2012) reported a 25-patient case series, in which superselective embolization applied to manage visceral pseudoaneurysms was associated with a hemorrhage recurrence rate of 15.8%; the authors concluded that the superselective approach was effective. However, reviewing this case series revealed that the culprit lesions contain various visceral splanchnic arteries. Therefore, we presumed that the hemorrhage recurrence rate would be higher if only the hepatic arteries and GDA are considered. To compare destructive and superselective approaches, Hur et al. (2011) determined hemorrhage recurrence rates and major complications in 16 patients with delayed PPH and GDA pseudoaneurysms. Superselective embolization was associated with a 100% hemorrhage recurrence rate and higher incidence of major complications (100%) compared with hepatic artery sacrifice (15.4%). Furthermore, a case series by Gwon et al. (2011) revealed a higher hemorrhage recurrent rate of superselective embolization in managing extrahepatic artery pseudoaneurysms. In our study, all four patients who underwent superselective embolization for a GDA pseudoaneurysm had recurrent hemorrhage. By contrast, in the hepatic artery sacrifice group, 2 (18%) of the 11 patients had recurrent hemorrhage, indicating a lower incidence rate and a rebleeding rate comparable to that in other studies (Biondetti et al. 2019; Floortje van Oosten et al. 2019). Therefore, our results support the finding that the destructive approach is more effective than superselective embolization in managing delayed PPH.

Hepatic artery sacrifice may cause severe hepatic complications due to hepatic flow occlusion. Stentgraft implantation has been revealed to be feasible and effective for treating visceral aneurysms and pseudoaneurysms while preserving vascular patency (Gwon et al. 2011; Pedersoli et al. 2016; Venturini et al. 2017; Schaarschmidt et al. 2018; Venturini et al. 2018). For delayed PPH, earlier case series have shown the effectiveness of covered stents for hemostasis (Hankins et al. 2009; Lovecek et al. 2014). Studies on small cohorts have determined that covered stent placement was associated with lower hemorrhage recurrence and hepatic complication rates compared with hepatic artery sacrifice or superselective embolization; these studies have thus recommended covered stent placement as a first-line treatment approach for delayed PPH (Huo et al. 2015; Gaudon et al. 2016). However, studies reporting follow-up results have shown that the 30-day mortality and hemorrhage recurrence rates associated with covered stent placement were not superior to those associated with embolization approaches (Ching et al. 2016; Hasegawa et al. 2017). Hassold et al. (2016) revealed that covered stents were associated with lower but not significant 30-day, 1-year, and 2-year survival rates than those of overall superselective and destructive embolization. Herein, we reported the data of only three patients receiving covered stent placement. The case number was small, and the hemorrhage recurrence, hepatic complication, and mortality rates associated with covered stent placement seemed similar to those associated with hepatic artery sacrifice. Because of the aforementioned mortality and morbidities from our limited experience, the long-term result of covered stent placement remains uncertain, despite achieving efficient short-term hemostatic results. Although the retrospective period of this study spanned > 15 years, covered stent placement was performed in later years because of the advancement of instruments and commercial availability. Its technical and clinical outcomes may improve after the accumulation of experience. Currently, we consider covered stent placement a reasonable alternative treatment, but it is not superior to hepatic artery sacrifice. Either hepatic artery sacrifice or covered stent placement can be chosen based on the experiences of duty doctors and instrument availability.

Notably, we found that the recurrent bleeding sites in patients receiving hepatic artery trapping and superselective embolization were the same as their previous bleeding sites. We managed the rebleeding by using additional coils at the hepatic artery. By contrast, the rebleeding sites for the covered stent group were different from the first site. Ching et al. (2016) used covered stents (65.7%) more often than they did coil embolization (23.6%) for hemostasis and found that recurrent bleeding mostly occurred from new sites of vascular injury rather than from the previously treated sites. We presumed that recurrent bleeding at the same site after embolization could be explained by unhealing ruptured vessels or by the rupture of vessels that recanalized with a larger diameter after the shock episodes of the first EVT had passed, making the previous coils insufficient to prevent rebleeding. Because the etiology of delayed PPH is anastomotic infection and leakage (Biondetti et al. 2019), we supposed that ongoing infection beyond the stent graft would continuously erode the vessels and result in rebleeding at different locations.

MDCT has been described as an essential tool for detecting bleeding in delayed PPH and other postoperative bleeding conditions (Chatani et al. 2018; Biondetti et al. 2019). According to our experience, MDCT may be helpful as a tailored treatment strategy for individual patients. For instance, the feasibility of covered stent placement is influenced by the tortuosity and size of the parent artery, and thin-section MDCT may be helpful in the planning stage because of its multiplanar reformation. To treat visceral artery aneurysms, Venturini et al. (2018) changed some patients’ treatments from a transfemoral approach to a transaxillary approach to increase the feasibility of stent placement after reviewing preinterventional CT results. MDCT can also provide information regarding extraluminal conditions, which may aid in determining the undamaged segment of the culprit vessel. Thus, routine use of MDCT before EVT in patients with delayed PPH may enhance treatment outcomes. Further research is required to support this strategy.

The present study has several limitations. First, the sample sizes for each treatment approach were small; in particular, only three patients received covered stent placement. Second, this study included a retrospective series covering a long study period. Thus, selection bias may have been involved; moreover, the possibility of missing data also existed. Third, three (75%) of four patients with recurrent hemorrhage who received superselective embolization eventually received hepatic artery sacrifice in their second treatments. Therefore, hepatic complications and mortality rates may overlap with hepatic artery sacrifice. Further research with larger sample sizes and standardized treatment algorithms are warranted.

## Conclusion

For managing delayed PPH through EVT, hepatic artery sacrifice was more effective than superselective pseudoaneurysm embolization because it was associated with lower hemorrhage recurrence rates. Covered stent placement and hepatic artery trapping had similar effectiveness. Interventional radiologists can select either hepatic artery sacrifice or covered stent placement to manage delayed PPH.

## Data Availability

The datasets used and/or analysed during the current study are available from the corresponding author on reasonable request.

## Abbreviations

CT: Computed tomography
EVT: Endovascular treatment
GDA: Gastroduodenal artery
MDCT: Multidetector computed tomography
NBCA: N-butylcyanoacrylate
PPH: Postpancreatectomy hemorrhage
PPPD: Pylorus-preserving Pancreaticoduodenectomy
